# Editorial: Non-canonical pathways of psychiatric drugs: beyond their neurotransmitter action

**DOI:** 10.3389/fnins.2023.1278748

**Published:** 2023-09-01

**Authors:** Hiram Tendilla-Beltrán, David Martín-Hernández

**Affiliations:** ^1^Instituto de Fisiología, Benemérita Universidad Autónoma de Puebla, Puebla, Mexico; ^2^Departamento de Farmacología y Toxicología, Facultad de Medicina, Instituto de Investigación Hospital 12 de Octubre (i+12), Instituto Universitario de Investigación en Neuroquímica, Universidad Complutense de Madrid, Madrid, Spain; ^3^Centro de Investigación Biomédica en Red de Salud Mental, Instituto de Salud Carlos III, Madrid, Spain

**Keywords:** psychiatric drugs, immunomodulation, antioxidant, neuroplasticity, epigenetics, microbiome

Psychiatric diseases are the leading global cause of disability and seriously impact society, the economy, and public health. A diverse array of treatments can lessen the agony endured by psychiatric patients, but a sizable minority of patients do not benefit from them. The underlying pathophysiology of psychiatric diseases is still poorly understood, while most of available pharmaceutical medications directly target neurotransmission systems originally associated with mental etiology.

Several studies have described the ability of psychiatric medications to modulate systems beyond the central nervous system (CNS), endangering effects spanning immune response, antioxidant system, neuroplasticity, epigenetics, and the microbiome. However, the current predominance of serotonergic, dopaminergic, GABAergic, and lithium-based medications stems from serendipity and experimental evidence. Unraveling their different actions beyond neurotransmission could shed light on novel mechanistic avenues for treatment development while deciphering relevant elusive features of psychiatric pathophysiology.

Thus, this Research Topic focuses on the non-canonical effects of psychiatric drugs beyond their neurotransmitter modulation and is composed of four manuscripts (one Original Research and three Review Articles), seeking to delve into uncharted dimensions of psychiatric pharmacology.

Zong et al. performed an integral multi-omic analysis encompassing subcortical covariance network, the Allen Human Brain Atlas (AHBA) transcriptomic dataset, and peripheral DNA methylation (DNAm) in first-episode drug-naïve schizophrenia patients and after 8 weeks of atypical antipsychotic monotherapy with risperidone. The treatment response yielded two distinct subgroups, responders and non-responders, to comprehensively understand efficacy heterogeneity across individuals. Regarding psychotic symptoms, non-responder patients exhibited increased baseline structural covariance within the striatal-hippocampus-pallidum pathway compared to the treatment responders. In the context of these symptoms, imaging variances showed a spatial association with the expression of genes enriched in neurobiological processes and dopaminergic pathways. For disorganized symptoms, there were notable structural covariance differences between both groups in the striatal-hippocampus-pallidum-thalamus pathway. The AHBA genes spatially correlated with baseline brain measurements overlap with the 108 schizophrenia candidate loci defining 19 genes of interest related to neurobiological processes. The DNAm analysis of these genes was associated with antipsychotic response. These results provide insight into the anatomical and genetic abnormalities crucial to therapeutic responsiveness during the early stages of schizophrenia. Furthermore, they contribute to the advancement of precision psychiatry (Gómez-Carrillo et al., 2023) by suggesting that subcortical structural covariance and peripheral DNAm could serve as valuable markers for predicting antipsychotic therapy response in schizophrenia.

Palagini and Bianchini provided an insightful narrative review centered on pharmacotherapeutics for insomnia, focusing on sleep regulation, neuroplasticity, and stress-related systems. Insomnia is a sleep disorder associated with stress which affects around 20% of the worldwide adult population, and this prevalence has even increased in recent years due to the Coronavirus Disease 2019 (COVID-19) pandemic (Cheshmehzangi et al., 2022). Moreover, chronic stress led to neuroplasticity alterations in a myriad of conditions, including sleep disorders, with multiple sleep-related neuroplasticity mechanisms described (Nissen et al., 2021; Weiss and Donlea, 2022). In this manuscript, the authors discuss the effects of two groups of GABA_A_ receptor agonists: short-medium-acting hypnotic benzodiazepines and the benzodiazepine analogs known as *Z*-drugs. Amidst these compounds, two drugs raise favorable choices surpassing their efficacious hypnotic attributes: triazolam (a short-medium-acting benzodiazepine) and eszopiclone (*Z*-drug). This distinction arises from their dual capacity to modulate stress response in insomnia and enhance “physiological” sleep. Such effects bear potential long-term benefits since they would not modify the homeostatic sleep processes and functions while correcting the associated neuroplastic alterations. Evidence from pre-clinical models in rodents demonstrates that triazolam, eszopiclone, and zaleplon (another *Z*-drug) do not appear to modify neuroplasticity. As a concluding remark, the authors state that pharmacotherapy for sleep disorders should effectively control arousal, sleep processes, and stress-related systems while avoiding potential neuroplasticity impairments.

Gangopadhyay et al. contributed with a detailed narrative review on the pathophysiology of non-alcoholic fatty liver disease (NAFLD) and its progressive form, non-alcoholic steatohepatitis (NASH), alongside their association with mental illnesses. NAFLD is the most prevalent cause of chronic liver disease worldwide, having inherent metabolic and cardiovascular comorbidities arising from hepatic dysfunction. However, psychiatric pathologies also exhibit a high comorbidity rate due to multiple factors, including genetic, metabolic, inflammatory, and environmental components. NAFLD/NASH and psychiatric disorders share systemic inflammation as a common pathophysiological mechanism (Goldsmith et al., 2023). Thus, NAFLD/NASH might contribute to the onset of psychiatric diseases via mechanisms rooted in inflammation. Vice versa, in patients diagnosed with psychiatric disorders, environmental factors such as smoking, sedentarism, hypercaloric/high-fat diet, and exposure to psychiatric drugs increase the risk of NAFLD. Multiple psychiatric drugs, including selective serotonin (5-HT) reuptake inhibitors (SSRI) antidepressants and atypical antipsychotics, are associated with an increased risk for NAFLD/NASH by multiple mechanisms, such as impairments in monoaminergic neurotransmission, insulin signaling, and iron metabolism. This information highlights the need to include metabolic liver disease-related recommendations in clinical guidelines for treating patients diagnosed with psychiatric diseases due to their heightened vulnerability to NAFLD/NASH.

Finally, Machado-Vieira et al. reviewed some non-canonical pathways pertinent to bipolar disorder (BD) pathophysiology. BD, a psychiatric disease characterized by extreme mood swings ranging from manic to depressive episodes, affects more than 1% of the global population and has a polygenic etiology (Rowland and Marwaha, 2018). Thus, multiple molecular and cellular alterations compromise brain function, including impaired neuroplasticity (at pre- and postsynaptic levels), mitochondrial dysfunction, oxidative stress, altered neurotransmission of monoamines and glutamate, poor neurotrophic factor activity, and inflammatory imbalance. The revision deals with the previously mentioned neurobiological mechanisms in BD, underscoring their potential as biomarkers and viable targets for pharmacotherapy. Specifically, immune-response modulators, histone deacetylase (HDAC) inhibitors, and the purinergic P2X7 receptor (P2X7R) arise as promising pharmacological targets for BD treatment, mainly owing to their anti-inflammatory properties, but also their neuroplasticity properties. The insights and debates addressed in this manuscript advance the quest for precision psychiatry (Gómez-Carrillo et al., 2023), which requires that both diagnosis and therapy go beyond the traditional neurotransmitter hypothesis for BD.

By improving our knowledge of the pathophysiological mechanisms and pharmacological targets that extend further than monoaminergic pathways, this Research Topic demonstrates the significance of non-canonical avenues in advancing the diagnosis and pharmacotherapy of psychiatric disorders.

## Author contributions

HT-B: Writing—original draft. DM-H: Writing—review and editing.
